# Selective Blockade of the Metabotropic Glutamate Receptor mGluR5 Protects Mouse Livers in In Vitro and Ex Vivo Models of Ischemia Reperfusion Injury

**DOI:** 10.3390/ijms19020314

**Published:** 2018-01-23

**Authors:** Andrea Ferrigno, Clarissa Berardo, Laura Giuseppina Di Pasqua, Veronica Siciliano, Plinio Richelmi, Ferdinando Nicoletti, Mariapia Vairetti

**Affiliations:** 1 Department of Internal Medicine and Therapeutics, Cellular and Molecular Pharmacology and Toxicology Unit, University of Pavia, 27100 Pavia, Italy; clarissa.berardo01@universitadipavia.it (C.B.); lauragiuseppin.dipasqua01@universitadipavia.it (L.G.D.P.); veronica.siciliano01@universitadipavia.it (V.S.); plinio.richelmi@unipv.it (P.R.); mariapia.vairetti@unipv.it (M.V.); 2 Department of Physiology and Pharmacology, Sapienza University, 00185 Roma, Italy; nicoletti@neuromed.it; 3 I.R.C.C.S. Neuromed, 86077 Pozzilli, Italy

**Keywords:** mGluR5, MPEP, MTEP, fenobam, ischemia, liver, mice, liver transplantation, mitochondria, hepatocytes

## Abstract

2-Methyl-6-(phenylethynyl)pyridine (MPEP), a negative allosteric modulator of the metabotropic glutamate receptor (mGluR) 5, protects hepatocytes from ischemic injury. In astrocytes and microglia, MPEP depletes ATP. These findings seem to be self-contradictory, since ATP depletion is a fundamental stressor in ischemia. This study attempted to reconstruct the mechanism of MPEP-mediated ATP depletion and the consequences of ATP depletion on protection against ischemic injury. We compared the effects of MPEP and other mGluR5 negative modulators on ATP concentration when measured in rat hepatocytes and acellular solutions. We also evaluated the effects of mGluR5 blockade on viability in rat hepatocytes exposed to hypoxia. Furthermore, we studied the effects of MPEP treatment on mouse livers subjected to cold ischemia and warm ischemia reperfusion. We found that MPEP and 3-[(2-methyl-1,3-thiazol-4-yl)ethynyl]pyridine (MTEP) deplete ATP in hepatocytes and acellular solutions, unlike fenobam. This finding suggests that mGluR5s may not be involved, contrary to previous reports. MPEP, as well as MTEP and fenobam, improved hypoxic hepatocyte viability, suggesting that protection against ischemic injury is independent of ATP depletion. Significantly, MPEP protected mouse livers in two different ex vivo models of ischemia reperfusion injury, suggesting its possible protective deployment in the treatment of hepatic inflammatory conditions.

## 1. Introduction

Glutamate is one of the major excitatory neurotransmitters in the central nervous system, signaling through ionotropic receptors, which are glutamate-gated, fast-responding ion channels, and metabotropic receptors (mGluR), which are slower responders, able to activate an intracellular cascade. Eight different mGluRs have been classified into three groups, on the basis of gene-sequence homology and transduction mechanism. Group I includes mGluR1 and mGluR5, which are coupled to the inositol phosphate/diacylglycerol signaling pathway and induce intracellular calcium mobilization. On the contrary, mGluRs belonging to Group II (mGluR2 and mGluR3 subtypes) and Group III (mGluR4, mGluR6, mGlu7, and mGluR8 subtypes) inhibit the production of cyclic AMP [1,2]. 

Evidence of the presence of glutamate receptors in peripheral organs has been published in recent years and numerous studies have demonstrated the presence of mGluRs in peripheral cells, many of them not originating from neural crests [3]. mGluRs have been found in the pancreas [4], in the liver [5], in the stomach mucosa [6], in human testes [7], and in primate ovaries [8]. These peripheral mGluRs can be activated by non-synaptic glutamate, probably synthesized from α-oxo-glutarate, a byproduct of the Krebs cycle. Glutamate can be transported outside the cell, where it intervenes in cell-to-cell communication or self-regulation by activating the mGluRs [9].

Sureda et al. (1997) first demonstrated the presence of a metabotropic glutamate receptor in the liver, showing that the competitive agonists quisqualate and 1-Amino-1,3-dicarboxycyclopentane (ACPD) stimulated [^3^H]inositolmonophosphate ([^3^H]InsP) formation in primary rat hepatocytes [10]. The existence of the mGluR5 subtype in primary rat hepatocytes was demonstrated in two articles by Storto and colleagues, using Western blot and polymerase chain reaction (PCR) analysis. More interestingly, these papers showed that hypoxic hepatocytes released more glutamate, probably saturating and hyperactivating mGluR5. Furthermore, hypoxic rat hepatocytes treated with the mGluR5 negative modulator 2-methyl-6-(phenylethynyl)pyridine (MPEP) or hepatocytes from mice knock-out (KO) for mGluR5s, were more resistant to hypoxic injury, suggesting that the activation of mGluR5s by endogenous glutamate promotes the progression of hypoxic injury [5,11]. 

Recently, MPEP has been found to reduce ATP content in microglia cells and astrocytes; direct or indirect receptor-mediated mechanisms have been proposed by the authors [12,13]. This findings, are, apparently, in contrast with the ability of MPEP to protect against ischemic injury, since the failure of ATP formation is the fundamental stressor in ischemic injury [14]. Hence this study attempted (1) to verify these published data in rat hepatocytes; (2) to compare the ATP-depleting ability of MPEP, with other negative allosteric modulators (NAMs), such as: 3-((2-Methyl-4-thiazolyl)ethynyl)pyridine (MTEP) and fenobam, or orthosteric ligands, such as the agonist dihydroxyphenylglycine (DHPG) and the antagonist (*S*)-4-carboxyphenylglycine (CPG); (3) to understand the mechanism of MPEP-mediated ATP depletion and its possible impact in the protection from ischemic injury in vitro. Finally, the goal of the study was to verify, for the first time, whether MPEP is able to protect whole livers in murine models of cold or warm ischemia-reperfusion injury. We found that MPEP, MTEP, but not fenobam, DHPG, or CPG, deplete ATP in cells, mitochondrial suspensions, and acellular solutions without altering mitochondrial functionality. MPEP and MTEP showed good protection from ischemic injury in vitro despite their ability to deplete ATP. Finally, we showed, for the first time, that mouse livers treated with MPEP or from mice knocked out for mGluR5, are more resistant to both cold and warm ischemia-reperfusion injury.

## 2. Results

### 2.1. mGluR5 NAMs Protect Rat Hepatocytes from Ischemic Injury Regardless of Their Ability to Deplete ATP

#### 2.1.1. Effects of MPEP and MTEP on Cellular and Mitochondrial ATP Content

**Figure 1:**
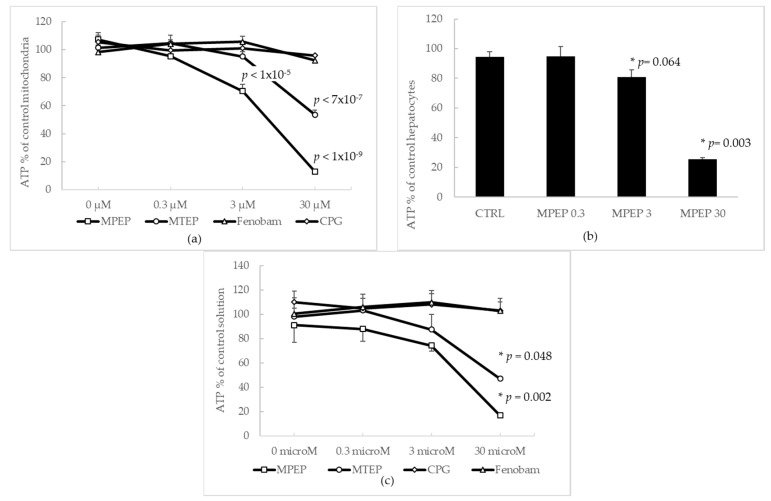
MPEP and MTEP deplete ATP from isolated mitochondria, primary hepatocytes, and acellular, ATP-containing solutions. (**a**) MPEP and MTEP reduce ATP content in a suspension of isolated hepatic mitochondria, in a dose dependent way, to 13.0 ± 1.3% and 53.7 ± 12.5% with respect to control mitochondria. Fenobam and CPG did not show any effect. DHPG did not alter ATP content [15]; (**b**) In primary rat liver hepatocytes, MPEP dose-dependently reduced ATP concentration, producing a near-significant effect at 3 µM and a markedly significant effect at 30 µM (*p* = 0.003). At 0.3 µM MPEP did not reduce ATP concentration in freshly isolated hepatocytes. DHPG did not alter ATP content [15]; (**c**) 10 µM ATP was added to a plain PBS buffer. ATP was significantly reduced by the addition of MPEP (16.8 ± 0.5% of control solution at 30 µM) and MTEP (47.1 ± 1.7% of control solution, at 30 µM) but not fenobam or CPG, reproducing the same trend observed in (**a**). The asterisk indicates a significant difference (Tukey’s Test) with respect to controls (0 µM).

ATP was measured in rat liver mitochondria in the presence of MPEP, MTEP, fenobam, CPG, and DHPG at different concentrations (0, 0.3, 3 and 30 µM). MPEP and MTEP reduced ATP content, in a dose-dependent way, to 13.0 ± 1.3% and 53.7 ± 12.5%, with respect to control mitochondria. Fenobam and CPG had no effect (Figure 1a) nor did DHPG alter ATP concentration [15]. In primary rat liver hepatocytes, MPEP dose-dependently reduced ATP concentration, producing a near-significant effect at 3 µM and a markedly significant effect at 30 µM (*p* = 0.003). At 0.3 µM MPEP did not reduce ATP concentration in freshly isolated hepatocytes (Figure 1b). When added to plain phosphate-buffered saline (PBS), 10 µM ATP was significantly reduced by the co-administration of MPEP (16.8 ± 0.5% of control solution at 30 µM) and MTEP (47.1 ± 1.7% of control solution, at 30 µM). Fenobam, CPG, and DHPG did not change ATP in PBS, reproducing the same trend observed in Figure 1a (Figure 1c). 

To rule out the possibility that MPEP might reduce ATP concentration in a receptor-dependent way, we evaluated ATP in hepatocyte extracts from mice KO for mGluR5, with respect to extracts from wild-type mice. No significant difference was found (20.03 ± 3.69 nmol/mL and 23.08 ± 6.63 nmol/mL in mGluR5 KO and wild type, respectively, *p* = 0.71).

#### 2.1.2. MPEP (30 µM) Does Not Alter Mitochondrial Functionality

**Figure 2:**
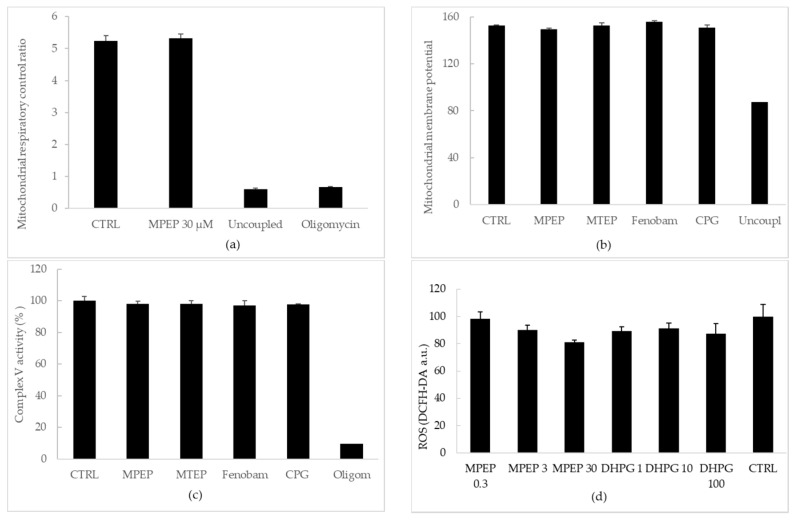
MPEP did not alter mitochondrial functionality. (**a**) MPEP 30 µM did not change respiratory control index; (**b**) MPEP 30 µM, MTEP 30 µM, fenobam 50 µM and CPG 100 µM did not alter mitochondrial membrane potential; (**c**) MPEP 30 µM, MTEP 30 µM, fenobam 30 µM and CPG 30 µM did not alter Complex V (FOF1-ATPase) activity with respect to control mitochondria; (**d**) MPEP and DHPG did not change mitochondrial ROS production with respect to controls. As negative controls mitochondria treated with olicomycin 2 µg/mL (Oligomycin) and/or subjected to three freeze/thaw cycles (Uncoupled) were used.

In order to exclude the possibility that MPEP and MTEP reduced mitochondrial functionality, mitochondria isolated from rat livers were assayed for respiratory control index (RCI), membrane potential, FOF1 ATPase activity and ROS production. When added to a mitochondrial suspension, MPEP 30 µM did not change RCI (Figure 2a). MPEP 30 µM, MTEP 30 µM, fenobam 30 µM and CPG 30 µM did not alter mitochondrial membrane potential and Complex V (FOF1ATPase) activity when tested with respect to control mitochondria (Figure 2b,c). Finally, different concentrations of MPEP and DHPG did not change mitochondrial ROS production with respect to controls. With increasing MPEP concentrations, there was a slight statistically insignificant decrease in ROS (Figure 2d). We do not consider this phenomenon to be associated with mitochondrial activity, since we observed no related changes in mitochondrial respiration or mitochondrial membrane potential.

#### 2.1.3. MPEP, MTEP and Fenobam Protect Rat Hepatocytes from Warm Ischemic Injury 

**Figure 3:**
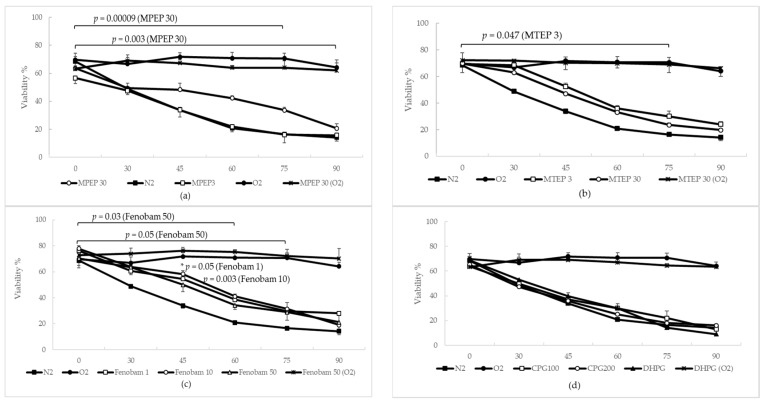
MPEP, MTEP and fenobam reduce mortality rate (measured by TB exclusion) of hypoxic primary hepatocytes. (**a**) The mortality rate of hepatocytes treated with MPEP 30 µM was lower than in control anoxic hepatocytes, in the 0′–90′ time range (*p* = 0.003, linear mixed-effects fitting analysis). The *p* value for this comparison increased in the 0′–75′ range (*p* = 0.00009, linear mixed-effects fitting analysis); (**b**) MTEP 3 µM reduced the mortality rate in this time range (*p* = 0.048, linear mixed-effects fitting analysis); (**c**) fenobam improved viability of hypoxic hepatocytes in both the 0′–60′ and 0′–75′ time range (*p* = 0.03 and 0.05, respectively, linear mixed-effects fitting analysis). Furthermore, fenobam 1 µM and 10 µM improved the vitality of hepatocytes at 45′ and 60′ (*p* = 0.05 and 0.003, respectively, Tukey’s Test); (**d**) the orthosteric antagonist CPG showed no protective effect. The administration of DHPG 100 µM–DFB 10 µM did not worsen cellular injury.

The mortality rate of hypoxic hepatocytes was evaluated by means of Trypan blue (TB) exclusion. MPEP, MTEP and fenobam reduced mortality rates with respect to untreated hypoxic hepatocytes. By comparing mortality curves by means of a linear mixed-effects fitting analysis, it emerged that, in the 0′–90′ time range, the mortality rate for hepatocytes treated with MPEP 30 µM was lower than in control anoxic hepatocytes (Figure 3a, *p* = 0.003). The *p* value for this comparison increased in the 0′–75′ interval (Figure 3a, *p* = 0.00009). A significant difference was also observed in this time range for anoxic hepatocytes treated with MPEP 3 µM (Figure 3b, *p* = 0.047) and fenobam 50 µM (Figure 3c, *p* = 0.05) with respect to untreated hypoxic hepatocytes. Furthermore, hepatocytes treated with fenobam 50 µM and MPEP 3 µM also had lower a mortality rate compared with anoxic controls in the 0′–60′ time range in which a significant difference was also observed for hepatocytes treated with MTEP 3 µM (*p* = 0.047). The orthosteric antagonist CPG showed no protective effect (Figure 3d). Administration of DHPG 100 µM-DFB 10 µM did not worsen cellular injury (Figure 3d), probably because, as previously demonstrated [5], mGluR5 receptors are already saturated with glutamate. Analyzing the 45′ time point by ANOVA, it emerged that, compared to anoxic controls, there was a statistical difference in the viability of cells treated with fenobam 1 µM and fenobam 10 µM, (Figure 3c, *p* = 0.05 and 0.003, respectively). The addition of MPEP 30 µM, MTEP 30 µM, fenobam 50 µM, or DHPG 100 µM to oxygenated hepatocytes did not produce any changes when compared to control oxygenated hepatocytes.

**Figure 4:**
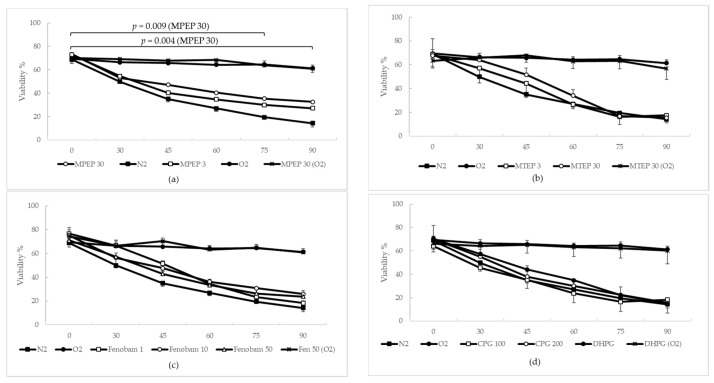
MPEP improves the viability (measured by LDH release) of hypoxic hepatocytes: (**a**) a statistical difference was observed in the 0′–90′ time range, between anoxic cells treated with MPEP 30 µM (*p* = 0.004, linear mixed-effects fitting analysis) and the corresponding untreated anoxic controls. The significance for this comparison decreased in the 0′–75′ time range (*p* = 0.009) and further decreased in the 0′–60′ time range (*p* = 0.048); (**b**) no statistical difference was observed, by means of linear mixed-effects fitting analysis or Tukey’s Test, for MTEP, although mortality rates showed a similar trend compared with that observed using TB exclusion; (**c**) no statistical difference was observed, by means of linear mixed-effects fitting analysis or Tukey’s Test, for fenobam, although mortality rates were similar to the rates observed using TB exclusion; (**d**) no statistical difference was observed using linear mixed-effects fitting analysis or Tukey’s Test for CPG, an orthosteric antagonist.

The mortality rate of hypoxic hepatocytes was also evaluated by means of lactate dehydrogenase (LDH) release. In the time range of 0′–90′, a statistical difference was observed between anoxic cells treated with MPEP 30 µM (*p* = 0.004, linear mixed-effects fitting analysis) and corresponding anoxic untreated controls. The significance for this comparison decreased in the 0′–75′ time range (*p* = 0.009) and further decreased in the 0′–60′ time range (*p* = 0.048) (Figure 4a). No further statistical difference was observed, although mortality rates had a similar trend compared with the one observed using TB exclusion (Figure 4b–d). The addition of MPEP 30 µM, MTEP 30 µM, fenobam 50 µM or DHPG 100 µM to oxygenated hepatocytes did not produce any changes when compared to control oxygenated hepatocytes.

#### 2.1.4. MPEP Prevents ATP Depletion in Hypoxic Hepatocytes

**Figure 5:**
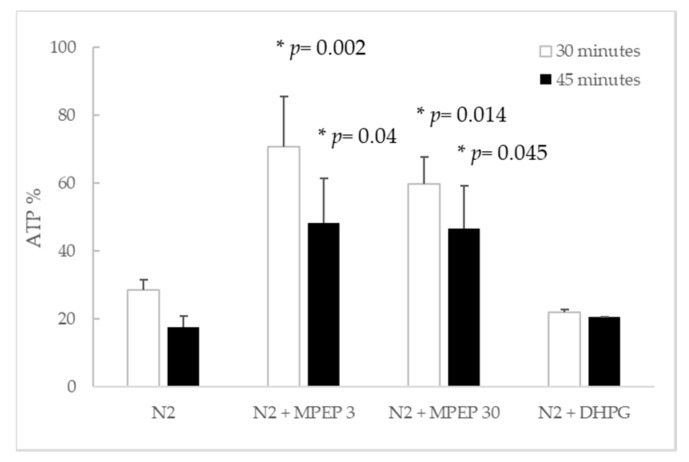
MPEP attenuates the time dependent ATP decrease induced by oxygen deprivation, 30′ and 45′ after N_2_ insufflation (Tukey’s Test).

Although ATP is depleted by MPEP and MTEP in normoxic hepatocytes, this tendency is reversed in hypoxic hepatocytes. In particular, hypoxic hepatocytes treated with MPEP 3 µM and 30 µM had higher ATP content than hypoxic controls, after 30′ and 45′ hypoxia. No statistical difference was observed for treatments with other mGluR5 ligands (Figure 5). 

### 2.2. MPEP Protects Mouse Livers in an Ex Vivo Model of Cold Storage/Reperfusion Injury

**Figure 6:**
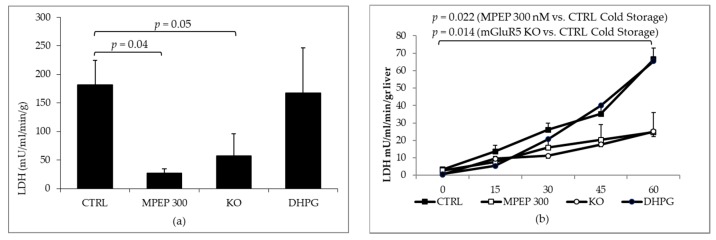
MPEP 300 nM reduced LDH release during cold storage and LDH release rate during warm reperfusion. (**a**) LDH was measured in the solution flushed out of the liver after 18 h cold storage. Control ischemic livers released significantly more LDH than livers preserved in MPEP containing solution and in livers from mGluR5 KO mice (*p* = 0.03 and 0.05, respectively, with Tukey’s Test). The addition of DHPG 100 µM and DFB 10 µM to the preservation solution produced no statistical difference vis-à-vis ischemic controls; (**b**) during oxygenated warm reperfusion, control ischemic livers had a significantly higher LDH release rate than livers treated with MPEP or livers from mGluR5 KO mice (*p* = 0.022 and 0.014, respectively, in a linear mixed-effects fitting analysis). When DHPG-DFB was added, the LDH release rate did not differ from ischemic controls.

Mouse livers were maintained for 18 h at 4 °C in Belzer preservation solution to simulate organ preservation for transplantation; livers were then reperfused with oxygenated Krebs–Henseleit buffer at 37 °C to simulate reperfusion and induce ischemia reperfusion injury. Livers treated with MPEP 300 nM (added to the preservation solution and the reperfusion solution) and livers from knock-out mice for mGluR5 showed a significant decrease in LDH release after cold preservation (Figure 6a) as well as during 60′ of oxygenated, normothermic reperfusion (Figure 6b). 

**Figure 7:**
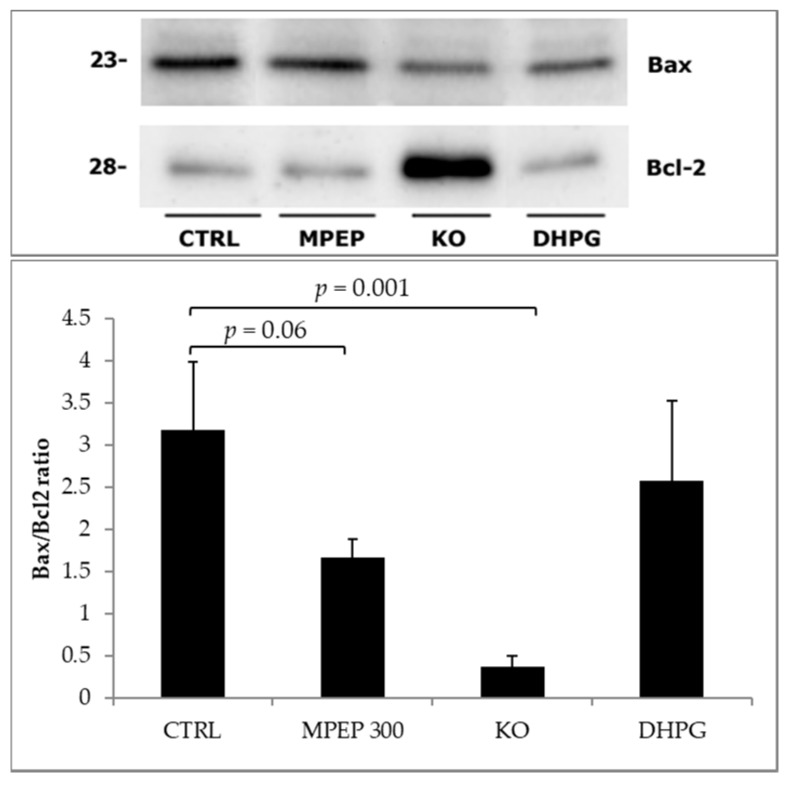
Tissue hepatic Bax/Bcl-2 ratio was significantly reduced in livers from mGluR5 KO mice (Tukey’s Test) after 18 h preservation by cold storage and 60′ warm reperfusion. Livers treated with MPEP during preservation and reperfusion had a near-significant reduction of Bax/Bcl-2 ratio, at the end of 60′ reperfusion.

At the end of 60′ warm reperfusion, the Bax/Bcl-2 ratio in livers from mice knock-out for mGluR5 was significantly lower than wild-type ischemic controls, whereas livers treated with 300 nM MPEP showed no significant tendency to decrease (Figure 7).

**Figure 8:**
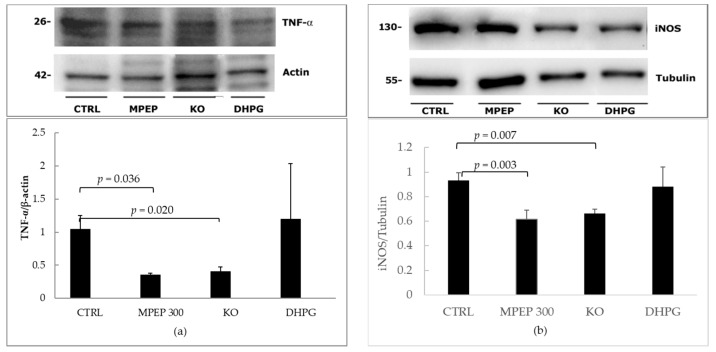

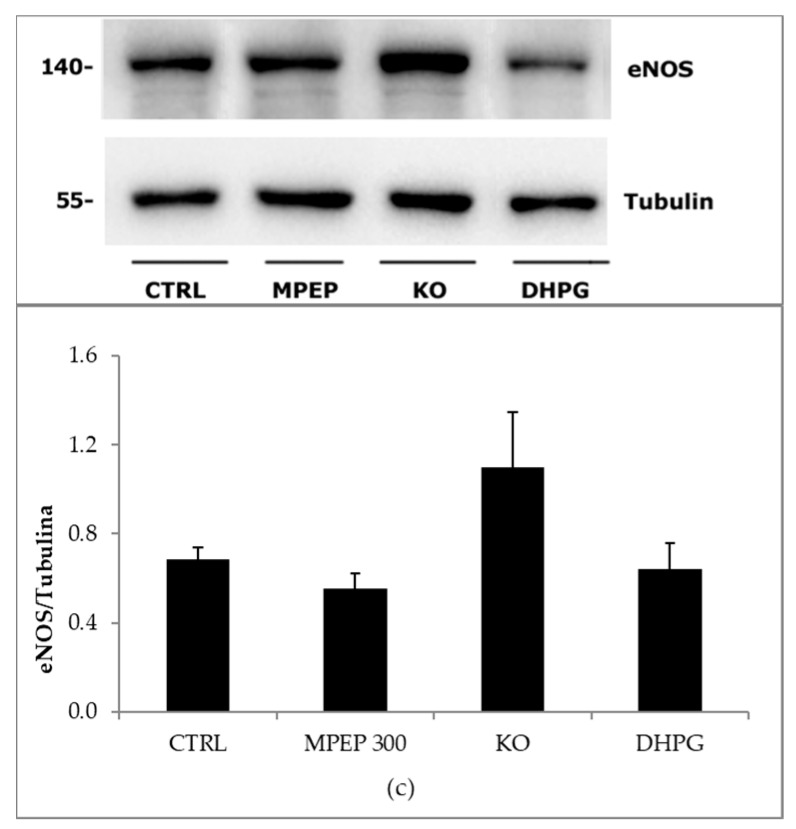
Hepatic expression of TNF-α, iNOS and eNOS in a model of cold storage/reperfusion injury. (**a**) Tissue hepatic TNF-α was significantly lower in livers from mGluR5 KO mice and MPEP treated livers (Tukey’s Test) after 18 h preservation by cold storage and 60′ warm reperfusion. Livers treated with DHPG during preservation and reperfusion showed no significant variation compared with control ischemic livers, at the end of 60′ reperfusion; (**b**) tissue hepatic iNOS was significantly lower in livers from mGluR5 KO mice and MPEP treated livers (Kruskal–Wallis non-parametric test and Dunn’s Test) after 18 h preservation by cold storage and 60′ warm reperfusion. Livers treated with DHPG during preservation and reperfusion showed no significant variation respect to control ischemic livers, at the end of 60′ reperfusion; (**c**) no significant difference was observed in eNOS expression at the end of 18 h cold storage and 60′ warm reperfusion, when comparing KO vs. CTRL groups (*p* = 0.08, ANOVA).

At the end of 18 h preservation and 60′ warm reperfusion, the expression of tumor necrosis factor-α (TNF-α) was significantly lower in livers from mGluR5 KO mice and in livers treated with MPEP 300 nM (Figure 8a). Similarly, the expression of inducible nitric oxide synthase (iNOS) was significantly lower in livers from mGluR5 KO mice and in livers treated with MPEP 300 nM (Figure 8b). On the contrary, no statistical difference was observed in the expression of endothelial nitric oxide synthase (eNOS), an endothelial enzyme involved in vasodilation (Figure 8c). 

ATP has been evaluated in extracts from livers collected at the end of oxygenated reperfusion. The trend of ATP reflected the level of injury or protection, with higher levels in livers treated with MPEP/KO livers and lower levels in livers treated with DHPG 100 µM. However, no significant difference was found (Table 1).

### 2.3. MPEP Protects Mouse Livers in an Ex Vivo Model of Warm Ischemia Reperfusion Injury

**Figure 9:**
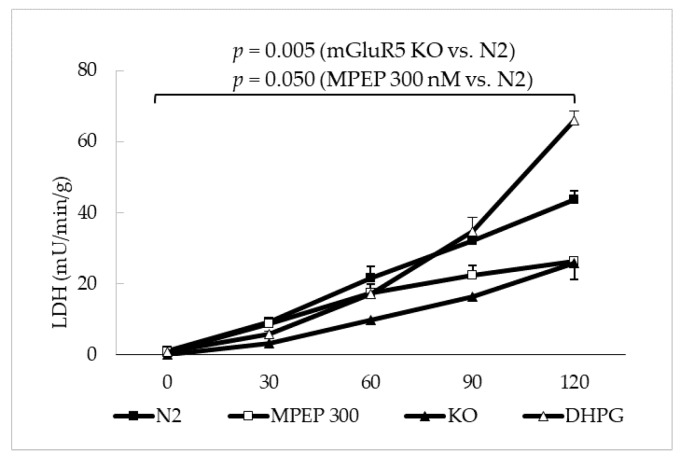
After 30′ perfusion with anoxic medium at 37 °C, during oxygenated warm reperfusion, the LDH release rate of control ischemic livers was significantly higher than livers treated with MPEP and livers from mGluR5 KO mice (*p* = 0.05 and 0.005, respectively, in a linear mixed-effects fitting analysis). The addition of DHPG-DFB was not associated to changes in LDH release rate when compared to ischemic controls.

**Figure 10:**
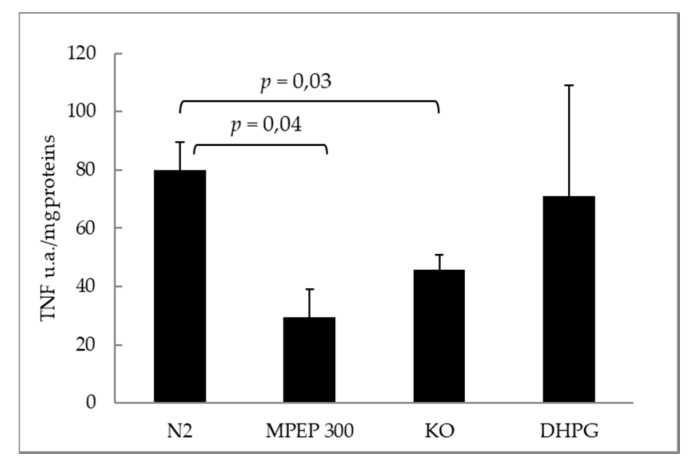
TNF-α release measured at the end of 120′ reperfusion was significantly lower in livers treated with MPEP and livers from mice KO for mGluR5 (Tukey’s Test).

Mouse livers were perfused with deoxygenated Krebs–Henseleit buffer at 37 °C for 30 min, then reperfused with oxygenated Krebs–Henseleit buffer at 37 °C for 120 min in order to induce warm ischemia/reperfusion injury. Livers treated with MPEP 300 nM (added to the perfusion solution at every stage of the experiment) and livers from knock-out mice for mGluR5 showed a significant decrease in the LDH release rate during 120′ of normothermic reperfusion (Figure 9). The administration of DHPG 100 µM and DFB 10 µM did not worsen ischemia/reperfusion injury in a significant way. The release of TNF-α in the perfusion solution, at the end of 120′ reperfusion, was lower in mGluR5 KO group and in the MPEP 300 nM group (Figure 10).

ATP has been evaluated in liver extracts at the end of 2 h anoxic perfusion. No significant difference was found (Table 2).

## 3. Discussion

In this work, we demonstrated for the first time that the pharmacological blockade of mGluR5 protects whole livers from ischemia/reperfusion injury in murine ex vivo models of cold and warm ischemia. Furthermore, we showed that MPEP, MTEP and fenobam share the ability to protect primary rat hepatocytes from ischemic injury, despite their differences with regard to ATP-depleting properties.

### 3.1. MPEP Sequesters ATP from Acellular Solution and Cellular Suspensions

The blockade of mGluR5 with MPEP 100 µM was recently described as the cause of a reduction in ATP content in murine BV-2 microglia cells. Since the blockade of mGluR5 in microglia is associated with an increase in Ca^2+^ release, the opening of the mitochondrial membrane permeability transition pore, mitochondrial membrane depolarization and increase in ROS production, the authors posited that, ATP depletion in BV-2 cells might be due to overworked ATPases during Ca^2+^ reuptake to the ER [12]. Furthermore, in astrocytes, the activation of a glutamate receptor was correlated to ATP release: 100 µM glutamate caused an increase in ATP up to 0.3–0.4 pmol/min [16]; on the other hand, MPEP completely inhibited the glutamate-evoked increase in extracellular ATP. This effect was not attributed to the activation/blockade of mGluR5, as mature astrocytes do not express this receptor; however, the authors posited involvement of the kainate receptor glutamate receptor, ionotropic kainate 2 (GluK2) [13]. We found that addition of MPEP 3–30 µM reduces ATP concentration when added to suspended rat hepatocytes’ mitochondria and acellular buffers. We also found that the addition of MTEP, which only differs from MPEP through the substitution of a benzene ring with a tiazole group, reduces the ATP content in mitochondria and buffered solutions, albeit to a lower extent. Both MPEP and MTEP are aromatic alkynes characterized by the presence of two aromatic rings joined together by a triple bond. On the contrary, fenobam and CPG, which do not possess an alkyne triple bond, do not sequester ATP either in acellular solutions or in cellular suspensions. Our findings suggest that the potential role of glutamate receptors in MPEP- and MTEP-associated ATP depletion, should be reconsidered, in view of the fact that MPEP and MTEP dose-dependently sequester ATP in acellular solutions at concentrations equal to or above 3 µM. 

### 3.2. NAMs Protect Hepatocytes from Ischemic Injury, Regardless of Their Ability to Induce ATP Depletion

Considering the importance of ATP in the progression of ischemic injury, we assessed whether MPEP, MTEP, Fenobam and CPG had different propensities as regards protecting rat hepatocytes from warm ischemia/reperfusion injury in vitro. We found that the blockade of mGluR5 protects from ischemia, independently of any ATP-depleting ability. Surprisingly, the most significant protection was found for cells treated with MPEP 30 µM (*p* = 0.00009 in the time range of 0′–75′). CPG did not reduce the mortality rate of ischemic hepatocytes, although we think that the tested CPG concentration (100 and 200 µM) was too low. In fact, CPG is an orthosteric antagonist with very low potency compared to MPEP, MTEP and fenobam; its IC50 in HEK-293 cells was estimated to be 1970 µM [17], compared to the IC50 of 36 nM found for MPEP [18]. In these experiments, the addition of DHPG 100 µM–DFB 10 µM did not significantly change mortality rates for hypoxic hepatocytes as compared with hypoxic control cells [15]. Similar findings are found in literature and have been justified in terms of excessive release of glutamate during ischemia, causing the saturation of mGluR5 [5]. 

Glutamate is the primary excitatory neurotransmitter in the CNS, playing a key role in cell excitotoxicity, via activation of glutamate receptors. In ischemic tissues, sustained cell membrane depolarization causes increased glutamate release; simultaneously, the process of glutamate reuptake is suppressed. The activation of glutamate receptors is a major route for excessive Ca^2+^ influx, believed to trigger excitotoxicity, a process responsible for acute neuronal death [19,20]. In particular, the activation of mGluR5 is linked to the formation of inositoltrisphosphate and diacylglycerole, which in turn induce oscillatory increases in cytosolic Ca^2+^ [21]. These intracellular changes are known to produce mitochondrial dysfunction, DNA fragmentation and oxidative stress, finally leading to cell death, especially in cells subjected to stress stimuli such as ischemia or hypoxia [22].

High glutamate and Ca^2+^ release have been previously associated to ischemic injury, in primary hepatocytes. An increase in extracellular glutamate release was observed in primary hepatocytes after 2 h warm ischemia. Blocking mGluR5s with the administration of MPEP 30 µM protected ischemic rat hepatocytes, suggesting that overactivation of glutamate receptors promotes progression of ischemic injury [23]. Furthermore, hepatocytes from mice knocked out for mGluR5s were more resistant to warm ischemia compared to control hepatocytes from wild-type mice [11]. 

In a paper by Elimadi and colleagues (2001), rat hepatocytes showed a significant increase in cytosolic Ca^2+^, in an in vitro model of cold ischemia/reperfusion. Primary rat hepatocytes were preserved in Belzer solution at 4 °C for 0, 24 and 48 h, then reoxygenated at 37 °C for 1 h. Pretreatment of hepatocytes with *m*-iodobenzylguanidine, which stabilizes Ca^2+^ homeostasis, improved hepatocyte viability [24]. 

Here we showed for the first time that MTEP and fenobam, well known selective mGluR5 negative allosteric modulators, protect rat hepatocytes from ischemia, probably by blocking part of the cytotoxic injury triggered by the excessive glutamate release. We also demonstrated that this protection does not seem to be affected by the ATP-depleting properties that MPEP and MTEP share. Surprisingly, the administration of MPEP 3 µM and 30 µM to hypoxic hepatocytes attenuated the hypoxia-driven ATP depletion. These data, insofar as they appear to be contradictory, suggest that, among the different variables affecting cellular ATP content during ischemia, including MPEP receptor-independent ATP depletion, lack of oxygen and MPEP receptor-dependent protection from ischemia, the prevailing variable may be MPEP protection from ischemic injury, producing as a consequence the attenuation of ATP depletion.

### 3.3. The Blockade of mGluR5 Protects Mouse Livers from Cold Ischemia and Warm Ischemia Reperfusion Injury

After inducing cold or warm ischemia in mouse livers, we evaluated hepatic conditions in a standard model of isolated perfused liver. Isolated perfused liver represents a suitable model when monitoring liver injury in rat and mouse livers [25,26] even in absence of hepatic artery reconstruction [27]. In this study, we found for the first time that mGluR5 blockade significantly reduces the LDH release rate, in both cold ischemia and warm ischemia reperfusion models. In livers preserved by cold storage, we found a significant decrease in LDH release rate, both at the end of 18-h preservation and during 60′ warm reperfusion, in MPEP and KO groups; we also found a reduction in TNF-α and iNOS expression, in KO and MPEP treated livers. The role of TNF-α has been widely studied in hepatic ischemia. Ischemic stress enhances the expression and release of proinflammatory molecules, such as TNF-α and iNOS; in addition, TNF-α can directly trigger apoptosis [28] and, thanks to its central role in promoting an inflammatory response, suppressing the formation of TNF-α might well be effective in attenuating acute post-ischemic inflammation and injury [29,30]. The expression of TNF-α and iNOS seems to be connected: the cloning of the promoter region of the iNOS gene showed the presence of TNF responsive elements [31]. Moreover, in a model of indomethacin-induced jejunoileitis in rats, it has been shown that treatment with TNF antibody reduces iNOS expression, suggesting that TNF-α may directly modulate iNOS expression [32]. To our knowledge, no publication in the literature has demonstrated a direct link between mGluR5 blockade and TNF-α downregulation and a similar conclusion is surely not deducible from our data. More reasonably, mGluR5 blockade is probably involved in halting excitotoxic events triggered by mGluR5 hyperactivation. The pharmacological inhibition of mGluR5-mediate excitotoxicity might play a role in indirectly reducing TNF-α and iNOS expression by reducing the source of cellular stress. 

In conclusion, in this work, we confirmed for the first time in the whole liver, that mGluR5 plays a key role in the ischemic injury progression. Furthermore, we showed that various mGluR5 NAMs share the ability to protect primary rat hepatocytes from ischemic injury; this protective effect was more significant for MPEP 30 µM, despite its ability to deplete cellular ATP. The mechanism underlying protection against ischemic injury reasonably consists in the block of cytotoxic events triggered by the overactivation of mGluR5s, such as the increase of Ca^2+^ release and the consequent mitochondrial dysfunction. The role of this receptor in liver has to be fully explored, although our data suggest their possible protective deployment in liver ischemic and inflammatory injury.

## 4. Materials and Methods

Original studies in animals have been carried out in accordance with a protocol approved by the local committee for animal welfare and MIUR, the Italian Ministry for University and Research, according to the strictest European Directives. 

MTEP and MPEP were purchased from Tocris Cookson Ltd. (Bristol, UK). All other chemicals used for experiments were of analytical grade and were purchased from Sigma (Milan, Italy). 

### 4.1. In Vitro Studies

#### 4.1.1. Mitochondrial Isolation and Assays

Mitochondrial isolation and assays: whole rat livers (9–15 g) were washed with ice-cold saline and processed immediately for mitochondria isolation. Minced tissue was homogenized in ice cold medium containing 0.25 M sucrose, 1 mM EDTA, 5 mM HEPES (pH 7.2) using a Teflon/glass Potter homogenizer (B. Braun, Melsungen, Germany). The homogenate was centrifuged at 1000× *g* for 10 min. The supernatant was centrifuged for 10 min at 10,000× *g*. The resulting pellet was resuspended in 0.25 M sucrose, 5 mM HEPES and centrifuged again 10 min at 10,000× *g* [33,34]. Single mitochondrial preparations were obtained for each individual animal (*n* = 6). Protein concentration was determined using the Lowry method [35]. Mitochondrial suspensions added with mGluR5 ligands or vehicle, were assayed for: ATP, mitochondrial membrane potential, RCI and FOF1 ATPase activity. Mitochondrial membrane potential was assessed by measuring the uptake of the fluorescent dye rhodamine 123 as previously described [33,34]. Briefly: after 15′ treatment with mGluR5 modulators, mitochondria were resuspended in a phosphate buffer with 0.3 μmol/L rhodamine 123. Rhodamine 123 fluorescence was monitored using a Perkin Elmer LS 50B fluorescence spectrometer at 503 (excitation wavelength) and 527 nm (emission wavelength). ΔΨ (mV) was calculated according to the following relationship: ΔΨ = −59 log (rhodamine 123) in/(rhodamine 123) out. Each measurement was performed in triplicate using fresh mitochondria preparations. RCI was measured using a Clarke electrode in a sealed mitochondrial chamber. Briefly, O_2_ consumption was measured in a sealed chamber at 25 °C by a Clark-type electrode. Mitochondria (1 mg/mL) were added to 1 mL of phosphate buffer containing 5 mM Mg^2+^ and 0.5 mM EGTA. Mitochondrial respiration was initiated with the addition of 10 mM succinate and 1 µM rotenone and oxidative phosphorylation was initiated with the addition of 5 mM adenosine diphosphate (ADP). O_2_ consumption recordings allowed the calculation of RCI, defined as the ratio between State 3 (maximum respiration rate reached in presence of respiratory substrates and ADP) and State 4 (low respiratory rate, respiratory substrates are present but ADP is exhausted) [36]. FOF1 ATPase activity was assayed using the Complex V MitoCheck kit from Cayman (Cayman Chemical, Ann Arbor, MI, USA). ROS formation was monitored by preloading mitochondria with 5 µM dichlorodihydrofluorescein diacetate (DCFH-DA). After 30′ mitochondria were withdrawn and centrifuged at 10,000× *g* for 10′. Pellets were resuspended in 0.5 mL KRH buffer and transferred to a 96-well plate for fluorescence measurements. Fluorescence values were measured on a Perkin Elmer Victor II (Perkin Elmer Inc., Waltham, MA, USA), using the excitation wavelength of 485 nm and emission wavelength of 530 nm [37].

#### 4.1.2. Hepatocyte Isolation

Hepatocytes were isolated from male Wistar rats (200–250 g, fasted for 18 h) by collagenase perfusion of the liver as previously described [38,39]. Hepatocytes were layered on top of a Percoll suspension and centrifuged at 500× *g* for 3 min. After removing the supernatant and the Percoll layer, cells were suspended in Krebs–Ringer–HEPES buffer containing 115 mmol/L NaCl, 5 mmol/L KCl, 2 mmol/L CaCl_2_, 1 mmol/L KH_2_PO_4_, 1.2 mmol/L MgSO_4_, and 2.5 mmol/L HEPES at pH 7.4 and incubated at 37 °C (final cell density, 10^6^/mL). 

#### 4.1.3. Warm Ischemia Experiments on Rat Hepatocytes

Anoxia was induced by blowing nitrogen for 3 min into hermetically sealed vials containing the cell suspension, while control vials were exposed to an oxygen-containing atmosphere. Hepatocytes were pre-incubated with the negative allosteric modulators MPEP (3 and 30 µM), MTEP (3 and 30 µM) and fenobam (1, 10 and 50 µM); with the orthosteric antagonist CPG (100 and 200 µM) and with the orthosteric agonist DHPG (100 µM) plus the mGluR5 selective positive allosteric modulator DFB (10 µM), for 15 min. The sealed vials were maintained at 37 °C in a water bath for 90′ and cell samples were collected at 0′, 30′, 45′, 60′, 75′ and 90′.

#### 4.1.4. Measurement of Cell Viability

Cell viability was monitored by TB exclusion [40] and by release of LDH into the medium, as described by Bergmeyer [41]. 

#### 4.1.5. Measurement of ATP

ATP variation in presence of mGluR5 modulators was evaluated in PBS added with ATP 10 µM, in mitochondria and in cell suspensions. ATP was measured by the luminescence method using the ATPlite luciferin/luciferase kit (Perkin Elmer Inc., Waltham, MA, USA). Luminescence was evaluated on a Perkin Elmer Victor II, using a white 96-well plate. 

### 4.2. Ex Vivo Studies

#### 4.2.1. Cold Ischemia Reperfusion Model

Livers were isolated from Balb c wild-type mice and mGluR5 knock-out mice, washed with Belzer cold preservation solution and stored on ice for 18 h, then reperfused with oxygenated Krebs–Henseleit at 37 °C for 1 h. After 18 h preservation, livers were washed with 5 mL of Krebs–Henseleit and the outflowing perfusate was stored at −80 °C for further analysis. Samples of the reperfusion solution were collected at 0′, 15′, 30′, 45′, and 60′. At the end of reperfusion, liver samples were snap-frozen in liquid nitrogen for further analysis. MPEP 0.3 µM or dimethyl sulfoxide (DMSO) was added to the preservation solution during cold storage and to the Krebs–Henseleit buffer during oxygenated reperfusion.

#### 4.2.2. Warm Ischemia Model

Livers were isolated from Balb c, wild-type mice and mgluR5 knockout mice, washed with Krebs–Henseleit solution and perfused for 30′ with deoxygenated Krebs–Henseleit at 37 °C. Deoxygenated Krebs–Henseleit was obtained by bubbling the solution with N_2_CO_2_ (95–5%). At the end of the ischemic period, livers were reperfused with oxygenated Krebs–Henseleit solution at 37 °C for 2 h. Samples of the reperfusion solution were collected at 0′, 30′, 60′, 90′, and 120′. At the end of reperfusion, liver samples were snap-frozen in liquid nitrogen for further analysis. MPEP 30 µM or DMSO was added to the preservation solution during warm ischemia and during oxygenated reperfusion.

#### 4.2.3. Perfusate Analysis

Liver parenchyma viability was assessed by measuring the release of LDH into the effluent perfusate, as described by Bergmeyer [41]. 

#### 4.2.4. Western Blot Analysis

Liver biopsies were homogenized in an ice cold sodium dodecyl sulfate (SDS) lysis buffer (pH 7.4) containing 1 mM PMSF and a mixture of protease inhibitors. Equal amounts of proteins were suspended in a SDS–bromophenol blue reducing buffer with 20 mM 1,4-dithiothreitol (DTT). Protein electrophoresis was performed on 7.5% SDS polyacrylamide gels run on a minigel apparatus (BioRad, Mini Protean II Cell); gels were electroblotted on ImmunBlot polyvinylidene difluoride (PVDF) Membrane (Biorad Laboratories Inc., Hercules, CA, USA) for 2 h using a Wet/Tank blotting system (BioRad Mini Trans-blot Cell); membranes were blocked in tris-buffered saline (TBS) buffer containing 5% bovine serum albumin or blotting grade blockers. Blots were then incubated at 4 °C overnight with primary antibodies all at 1:1000 dilution, with the exception of anti-actin (1:10,000). As primary antibodies we used: mouse monoclonal anti-Tubulin (Sigma Aldrich, Milan, Italy); goat polyclonal anti-TNF-α, rabbit polyclonal anti-Bax, mouse monoclonal anti-Bcl-2, rabbit polyclonal anti-eNOS and rabbit polyclonal anti-actin (Santa Cruz Biotechnology, Dallas, TX, USA), rabbit polyclonal anti-iNOS (Cayman chemical, Ann Arbor, MI, USA). Specific peroxidase-conjugated anti-IgG antibodies were from Santa Cruz Biotechnology. Blots were incubated for 1 h with secondary antibodies (peroxidase-coupled anti-mouse and anti-rabbit, Biorad) diluted 1:2000 with PBS. Immunostaining was revealed by ECL using a Chemidoc XRS Plus (Biorad Laboratories Inc., Hercules, CA, USA) [42]. 

#### 4.2.5. Statistical Analysis

Statistical analysis was performed by means of R Statistical software (v. 3.3.0) and the graphical interface R Studio (v. 1.0.143). The normality and homogeneity of variances were verified using Shapiro’s test and Levene’s test, respectively. In the majority of cases, data had normal distribution curves, where analyzed with ANOVA, followed by Tukey’s HSD Test for multiple comparisons. The Kruskal–Wallis non-parametric test was used for iNOS data since they had dishomogeneous variances. Dunn’s test was used for multiple comparisons. With measurements repeated against different time points, significance was analyzed by fitting data in a linear mixed-effects model.

## Figures and Tables

**Table 1:** ATP measured in liver extracts at the end of 1 h reperfusion (nmol/mg proteins). No significant difference was found among groups (ANOVA).

CTRL	MPEP 300 nM	KO	DHPG 100 µM
2.39 ± 0.34	3.52 ± 1.56	2.81 ± 0.92	1.82 ± 0.87

**Table 2:** ATP measured in liver extracts at the end of 2 h anoxic perfusion (nmol/mg proteins). No significant difference was found among groups (ANOVA).

CTRL	MPEP 300 nM	KO	DHPG 100 µM
2.48 ± 0.55	2.90 ± 0.70	2.44 ± 0.82	2.13 ± 0.77

## Abbreviations

[^3^H]InsP	[^3^H]inositolmonophosphate
ACPD	(1*S*,3*R*)-1-aminocyclopentane-1,3-dicarboxylic acid
ADP	adenosine diphosphate
DCFH-DA	dichlorodilhydrofluorescein diacetate
DFB	3,3′-difluorobenzaldazine
DMSO	dimethyl sulfoxide
DTT	1,4-dithiothreitol
eNOS	endothelial nitric oxide synthase
ER	endoplasmic reticulum
Gluk2	glutamate receptor, ionotropic kainate 2
iNOS	inducible nitric oxide synthase
KO	knock-out
PCR	polymerase chain reaction
PVDF	polyvinylidene difluoride
SDS	sodium dodecyl sulfate
TBS	tris-buffered saline
TNF-α	tumor necrosis factor-α
